# Dielectric Properties and Energy Storage Densities of Poly(vinylidenefluoride) Nanocomposite with Surface Hydroxylated Cube Shaped Ba_0.6_Sr_0.4_TiO_3_ Nanoparticles

**DOI:** 10.3390/polym8020045

**Published:** 2016-02-16

**Authors:** Shaohui Liu, Shaomei Xiu, Bo Shen, Jiwei Zhai, Ling Bing Kong

**Affiliations:** 1 Key Laboratory of Advanced Civil Engineering Materials of Ministry of Education, Functional Materials Research Laboratory, School of Materials Science & Engineering, Tongji University, 4800 Caoan Road, Shanghai 201804, China; qqliushaohui@163.com (S.L.); 1534890522@qq.com (S.X.); shenbo@tongji.edu.cn (B.S.); 2 School of Science, Henan Institute of Engineering, Zhengzhou 451191, China; 3 School of Materials Science and Engineering, Nanyang Technological University, 50 Nanyang Avenue, Singapore 639798, Singapore; ELBKong@ntu.edu.sg

**Keywords:** dielectric properties, inorganic–organic nanocomposite, surface hydroxylation, energy storage density

## Abstract

Ceramic-polymer nanocomposites, consisting of surface hydroxylated cube-shaped Ba_0.6_Sr_0.4_TiO_3_ nanoparticles (BST–NPs) as fillers and poly(vinylidenefluoride) (PVDF) as matrix, have been fabricated by using a solution casting method. The nanocomposites exhibited increased dielectric constant and improved breakdown strength. Dielectric constants of the nanocomposite with surface hydroxylated BST–NPs (BST–NPs–OH) were higher as compared with those of their untreated BST–NPs composites. The sample with 40 vol % BST–NPs–OH had a dielectric constant of 36 (1 kHz). Different theoretical models have been employed to predict the dielectric constants of the nanocomposites, in order to compare with the experimental data. The BST–NPs–OH/PVDF composites also exhibited higher breakdown strength than their BST–NP/PVDF counterparts. A maximal energy density of 3.9 J/cm^3^ was achieved in the composite with 5 vol % BST–NPs–OH. This hydroxylation strategy could be used as a reference to develop ceramic-polymer composite materials with enhanced dielectric properties and energy storage densities.

## 1. Introduction

Recently, a great deal of attention has been paid to developing high energy-storage density polymer-based capacitors, due to their potential applications in modern electronic and electrical power systems, such as electronic components, pulsed power sources and hybrid electric vehicles [1,2,3,4,5,6,7,8,9]. Compared with other electrical energy-storage devices, polymer-based capacitors have several advantages, such as fast charge/discharge (<1 μs), high working voltage, simple processing and cost-effectiveness. However, their energy densities are lower than that of electrochemical devices, such as batteries and double-layer super capacitors, by at least one order of magnitude. For example, the energy density of biaxially oriented polypropylene (BOPP), which is one of the most representative commercial polymer capacitor films, is only 1–2 J/cm^3^. As a result, developing high energy density polymer-based dielectric capacitors has become an active research topic in recent years [5,10,11,12,13,14].

The energy density of dielectric materials is defined as U=∫EdP, where *E* is applied electric field and *P* is polarization. To obtain high energy density, dielectric materials should have a high *E* and a high *P*. However, it is still a challenge to modulate the two parameters simultaneously. In addition, polymers have relatively low dielectric constant (e.g., <10). Although ferroelectric ceramics (e.g., BaTiO_3_) have high dielectric constant, their applications have been largely limited, due to their low breakdown strength and processing difficulty. In comparison, polymers, such as PVDF and BOPP, are flexible and easy fabrication. Moreover, polymers have much higher breakdown strength than ceramics. Therefore, nanocomposites have been considered to be a unique platform to combine the advantages of polymers (matrix) and ceramics (fillers), so as to achieve high energy density materials.

Ceramic fillers, such as Pb(Zr,Ti)O_3_, Ba_1__-x_Sr_x_TiO_3_ and BaTiO_3_, have been widely used to prepare polymer-ceramic composites with increased dielectric constant and enhanced energy density. However, a large quantity of the ceramic fillers is usually needed in order to achieve high dielectric constant. In this case, the breakdown strength and mechanical properties of the composites are seriously deteriorated [8].

Furthermore, it has been widely accepted that the introduction of ceramic fillers reduces the breakdown strength of composites, thus leading to low energy density. The reduction in breakdown strength of composites is mainly attributed to the weak interface interaction between the polymer matrix and ceramic fillers, owing to their poor chemical compatibilities. In this regard, many efforts have been made to improve the dispersing behaviors of ceramic fillers in polymer matrix. One strategy is to modify the surface of ceramic fillers so that a ceramic-polymer interfacial layer can be formed through chemical bonding. As a result, homogeneity of the nanocomposites will be greatly improved, which is essential for them to have excellent energy storage properties [1].

Additionally, particle shape of the ceramic filler also plays a decisive role in determining the energy storage density of polymer nanocomposites [15]. This is because fillers with different particle shapes have different surface areas and, thus, different interfacial areas in the nanocomposites, leading to different interfacial polarization and hence different energy storage properties. At the same time, connectivity of a nanocomposite is closely related to the particle shape of the fillers [16]. Extensive studies have been conducted on the synthesis and characterization of ceramic-polymer nanocomposites, containing Ba_x_Sr_1-x_TiO_3_ particles with different morphologies, including nanospheres [17], nanotubes [18], nanofibers [19], and nanowires [6]. Until now, there has been no report on PVDF-based nanocomposites with cube-shaped Ba_0.6_Sr_0.4_TiO_3_ nanoparticles (BST–NPs) and surface hydroxylation of BST–NP (BST–NPs–OH) as fillers.

In this work, dielectric and energy storage properties of PVDF nanocomposites with cube shaped BST–NPs–OH fillers were systematically studied. The cube-shaped BST–NPs were synthesized by using a molten salt method. BST–NPs–OH/PVDF nanocomposites were fabricated by using a solution casting method. The effect of content of the BST–NPs–OH on the microstructure, dielectric property and energy storage properties of the nanocomposites were investigated. Dielectric properties of nanocomposites have also been analyzed theoretically.

## 2. Experimental Section

Barium hydroxide (Ba(OH)_2_; 99%), strontium hydroxide (Sr(OH)_2_; 99%) and titanium dioxide (TiO_2_; 99%) were obtained from Alfa Aesar (Beijing, China). NaOH and KOH were purchased from Sinopharm Chemical Reagent Co., Ltd. (Shanghai, China). PVDF powders were purchased from 3F Co. (Shanghai, China). All chemicals were used as received without further purification.

Cube-shaped BST–NPs were synthesized by using a molten salt method, with NaOH–KOH mixture as the molten flux. High purity Ba(OH)_2_, Sr(OH)_2_ and TiO_2_ were used as raw materials to form Ba_0.6_Sr_0.4_TiO_3_. The raw materials were mixed with NaOH–KOH mixture. The mixtures were heated to 200 °C for 12 h. After reaction, the solidified melts were washed with distilled water at room temperature. The washed powders were then dried at 100 °C overnight in air. The final products were cube-shaped BST–NPs.

The BST–NPs were dispersed in an aqueous solution of H_2_O_2_ (35%, 350 mL), which were stirred and heated at 100 °C for 3 h. The suspensions were subsequently centrifuged at 3000 rpm for 10 min. The collected powder were washed with distilled water and ethanol and then dried at 80 °C for 12 h to obtain surface hydroxylated BSTs NP (BST–NP–OH).

PVDF powders (3F Co., China) were dissolved in dimethylformamide (DMF) first and then the BST–NPs–OH were introduced under constant stirring at 40 °C for 10 h to form stable suspensions. Volume fractions of BST–NPs–OH in the composites were varied from 0 to 40 vol %. Thin film samples were prepared by using tape-casting method with the suspensions on indium tin oxides (ITO) glass. The wet tapes were dried in vacuum at 60 °C for 10 h and then heated at 200 °C for 10 min, followed by quenching in ice-water. The samples were subsequently dried at 40 °C for 24 h. Thickness of the films was controlled in the range of 10–15 μm.

## 3. Characterization

X-ray diffraction (XRD) was used to study phase composition of the samples, with Cu-Kα radiation by using a RIGAKU D/max2550 diffractometer (Beijing, China). Fourier-transform infrared spectroscopy (FTIR) was recorded by using a Bruker Tensor 27 spectrometer (Ettlingen, Germany) over 450–4000 cm^−1^. X-ray photoelectron spectroscopy (XPS) was used to verify the surface-hydroxylated BST–NPs with Al Ka radiation (160 eV) using a Kratos Axis Ultra DLD multi-technique XPS equipment (Manchester, UK). Thermogravimetric analysis (TGA) was conducted using a using a NETZSCH STA449C instrument (Bavaria, Germany) at a heating rate of 10 °C·min^−1^ in N_2_ flow (20 mL·min^−1^). Microstructure of the samples was observed by using scanning electron microscopy (SEM, XL30FEG, Philips, The Netherlands) and transmission electron microscopy (TEM, CM200FEG, Philips, The Netherlands). For SEM measurement, a proper amount of BST-NPs power was dispersed in ethanol and then dropped onto the surface of Si wafer slides. The cross-section SEM of samples were prepared by fracturing the composites films in liquid nitrogen and the fractured surface was sputtered with thin layers of gold to avoid the accumulation of charge. Broadband frequency dielectric properties of the composites were measured by using a 4980A LCR meter (Agilent, Palo Alto, CA, USA) over 0.1–1000 kHz at various temperatures. DC breakdown strength was measured by using a breakdown strength tester (ENTAI, Nanjing, China) in silicone oil at room temperature (25 °C) by applying a DC voltage ramp at a rising rate of 200 V s^−1^ and a limit current of 5 mA. 10 samples were measured for each condition. The nanocomposite films were cut into square of 1 × 1 cm^2^, with a thickness of about 10 μm. The top gold electrodes with diameter of 2 mm and thickness of 40 nm were sputtered with a shadow mask, while bottom electrodes were sputtered without the use of shadow mask. Samples were placed between two stainless steel columnar electrodes (Ф = 1 mm). Polarization–electric field loops (*P-E*) were measured by using a Premier II ferroelectric test system in silicone oil to avoid electrical discharges.

## 4. Results and Discussion

**Figure 1:**
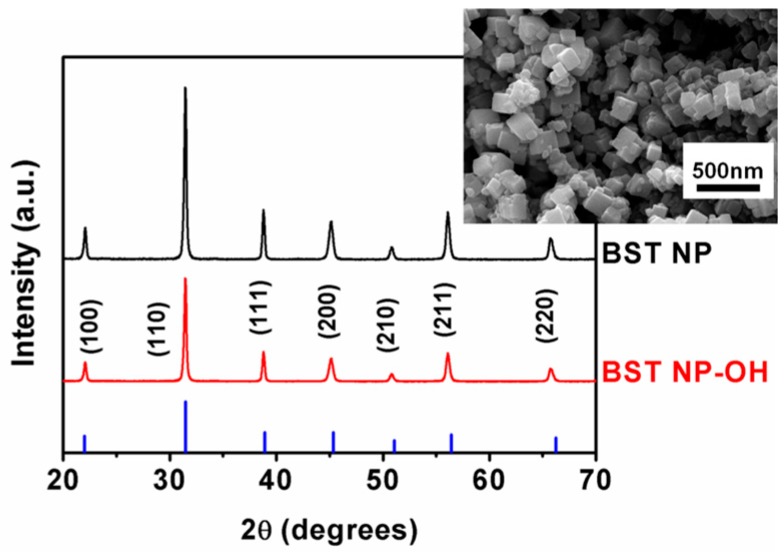
XRD patterns of the as-synthesized BST–NPs and BST–NPs–OH. The inset shows a SEM image of the BST–NPs.

Figure 1 shows XRD patterns of the as-synthesized BST–NPs and the BST–NPs–OH. It was shown that the strong peaks at 2θ that corresponded to 22° (100), 31° (110), 39° (111), 45° (200), 56° (211), and 66° (220) were assigned to BST with a perovskite structure (PDF#34-0411). No visible signal of the presence of secondary phases was observed. XRD results exhibit no changes in the sample of the crystal structure of both BST–NPs–OH and untreated BST–NPs. SEM image revealed that the BST–NPs have a faultless cubic morphology, with an average size (side length) of about 110 nm (inset of Figure 1).

**Figure 2:**
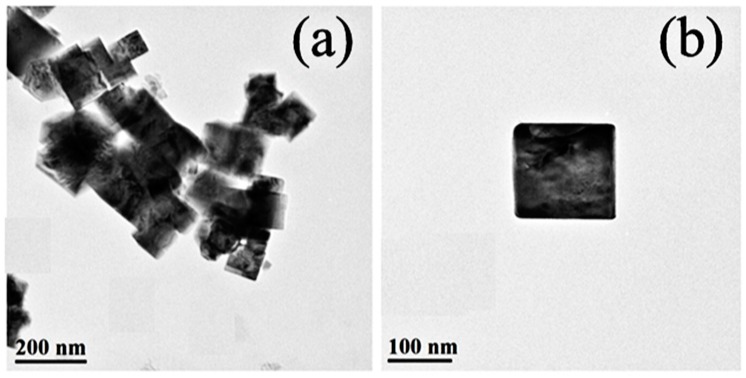
TEM images of the BST–NP at low (**a**) and high (**b**) magnifications.

The faultless cubic shape of the BST–NPs is confirmed by the TEM observation, as shown in Figure 2. The cubic nanoparticles have an average size of about 110 nm, in a good agreement with the SEM result.

**Figure 3:**
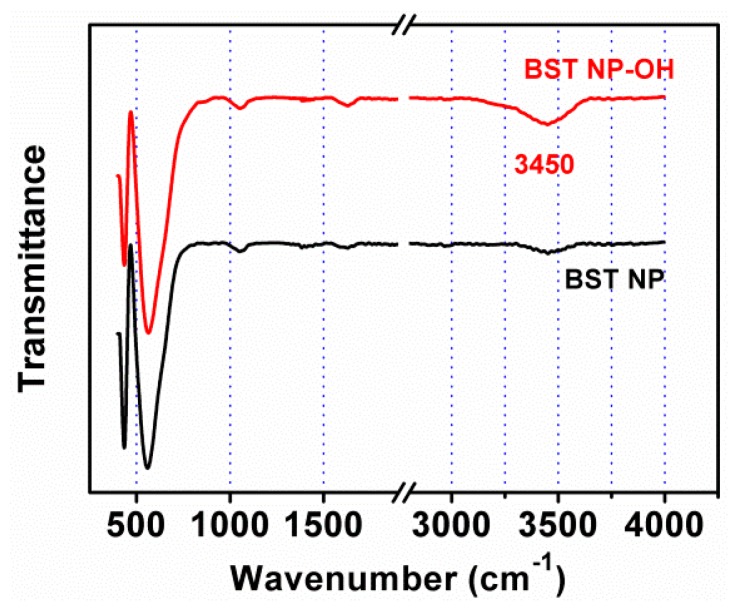
FT-IR spectra of the as-synthesized BST–NPs and the BST–NPs–OH.

**Figure 4:**
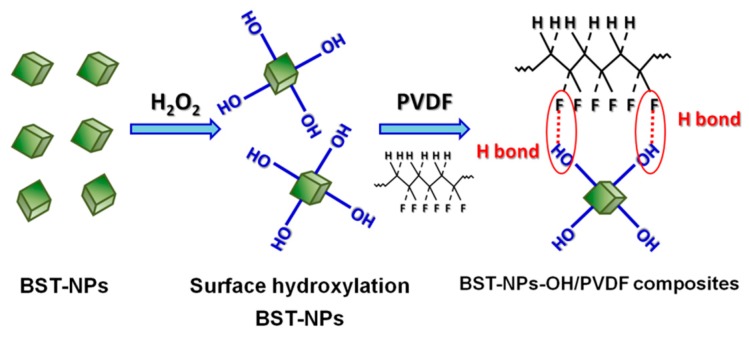
Schematic diagram illustrating hydroxylation of the BST–NPs and formation of a bond between the F atoms on the PVDF chains and the –OH groups on surface of the BST–NPs–OH.

Figure 3 shows FTIR spectra of the as-obtained BST–NPs and the BST–NPs–OH. The band at 550 cm^−1^ is associated to the bond vibration of Ti–O [20]. The new band at 3450 cm^−1^ corresponded to the stretcing mode of –OH [21], confirming the surface hydroxylation of the BST–NPs. The surface modification of the BST–NPs by the H_2_O_2_ are demonstrated to act as a bridge to between the F atoms on the PVDF and the –OH groups on the surface BST–NPs–OH (Figure 4).

**Figure 5:**
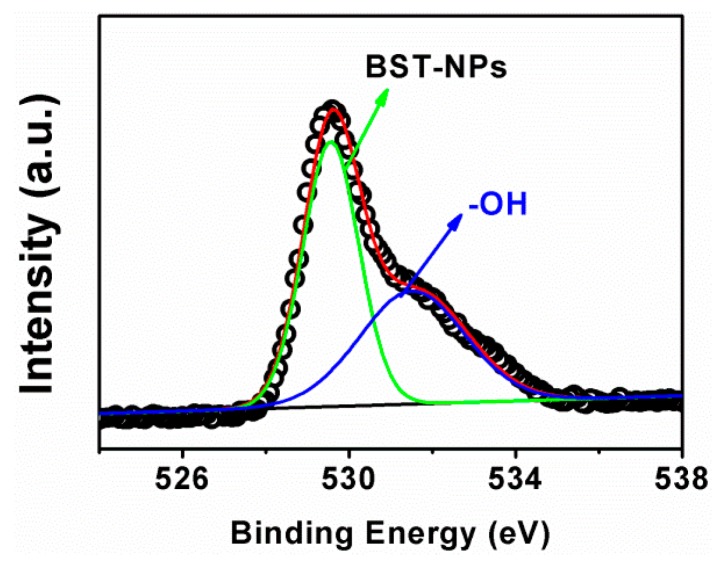
XPS spectra of O 1s of BST–NPs–OH.

Figure 5 shows the O1s spectra for BST–NPs–OH. We can see the peaks of O1s (529.4 and 531.5 eV) corresponding to the O atoms in Ba_0.6_Sr_0.4_TiO_3_ (O_–BST_) and free –OH (O_–OH_) [22], which confirming that the hydroxylate groups were introduced onto the surface of BST–NPs–OH.

**Figure 6:**
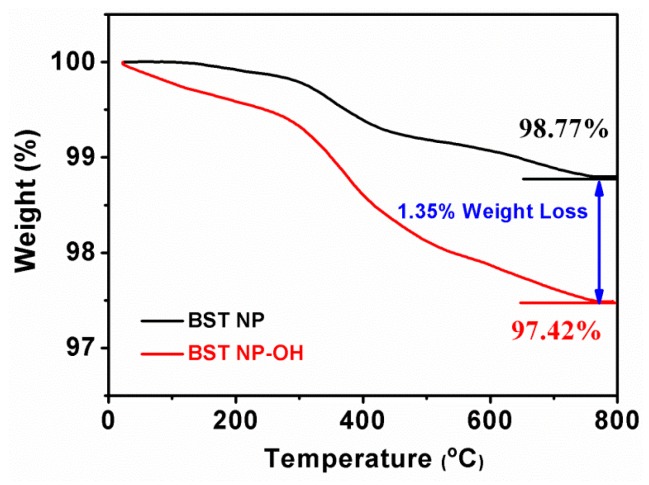
TGA curves of the untreated BST–NPs and the BST–NPs–OH.

TGA curves provided further evidence of the presence of the surface hydroxylation. A difference in the weight loss between the BST–NPs and BST–NPs–OH is obviously observed in TG curves, as shown in Figure 6. The weight loss of the BST–NPs–OH is larger than that of the BST–NPs by a value of 1.35% at 800 °C, which can be attribute to the vaporization of the hydroxyl groups. Moreover, the large weight loss before 300 °C of the BST–NPs–OH sample indirectly confirms that the hydroxylate groups were grafted onto the surface of BST–NPs [23].

**Figure 7:**
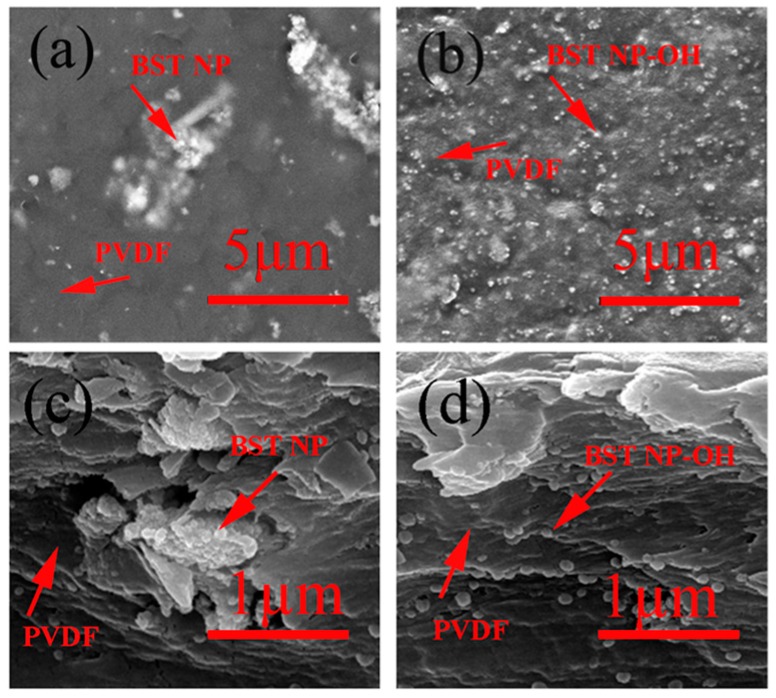
Surface SEM images of the PVDF nanocomposites: (**a**) BST-NPs/PVDF, (**b**) BST–NPs–OH/PVDF. Cross-section SEM of the PVDF nanocomposites: (**c**) BST-NPs/PVDF, (**d**) BST–NPs–OH/PVDF. All samples contain 10 vol % filler.

Figure 7 shows the surface SEM and cross-section SEM of BST–NPs/PVDF and BST–NPs–OH/PVDF nanocomposites (all the nanocomposites contain 10 vol % nanoparticles). It can be observed in Figure 7b,d that most of the BST–NPs–OH are well-dispersed in BST–NPs–OH/PVDF. However, in the BST–NPs/PVDF nanocomposite sample (Figure 7a,c), the aggregations of BST–NPs are clearly observed. Some voids and pores can be observed in BST–NPs/PVDF. The BST–NPs–OH/PVDF nanocomposites have hardly any small voids between the BST–NPs–OH and PVDF. This result indicates the surface hydroxylation could not only facilitate its dispersion in the polymer matrix but also strongly chain with the polymer matrix by hydroxyl bonds in the interface.

**Figure 8:**
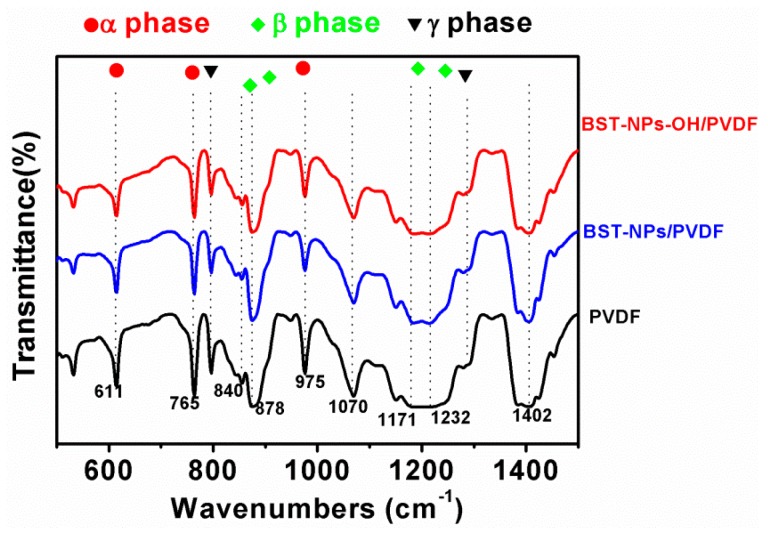
FT-IR spectra of the PVDF, BST–NPs/PVDF and BST–NPs–OH/PVDF nanocomposite films at a filler concentration of 10 vol %.

PVDF is a ferroelectric polymer, which has a complex structure and exhibits five crystalline phases, in which α, β, and γ are the most possible phases. Therefore, it is of importance to know the effects of fillers on the structure of the PVDF matrix. FT-IR techniques were used to obtain the structure information on PVDF in nanocomposites. The peaks of 840, 878, 1171, and 1232 cm^−1^ indicate the β-phase of PVDF, whereas the absorption bands at 611, 765, and 975 cm^−1^ indicate the α-phase of PVDF. The peaks of 794 and 1284 cm^−1^ indicate the γ-phase of PVDF. Figure 8 shows the FT-IR spectra of the PVDF, BST–NPs/PVDF and BST–NPs–OH/PVDF nanocomposite films at a filler concentration of 10 vol %. Each nanocomposite exhibits the characteristic absorption bands of α, β, and γ phases, indicating the PVDF matrix is composed of these three phases in the nanocomposites.

**Figure 9:**
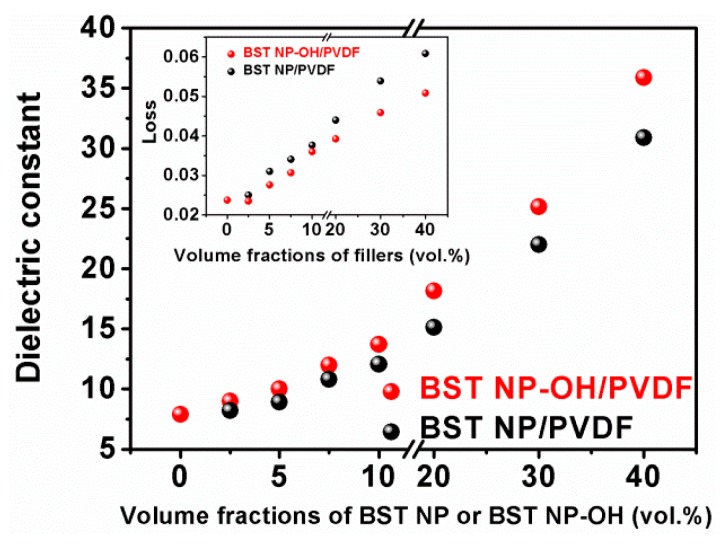
Dielectric constants of the BST–NPs/PVDF and BST–NPs–OH/PVDF nanocomposite films as a function of filler concentration measured at 1 kHz.

Figure 9 shows dielectric constants of the composites at 1 kHz as a function of the filler content. Pure PVDF has a relatively low dielectric constant of 7.9. In both composite samples, the dielectric constant gradually increases with increasing filler content. The same with 40 vol % BST–NPs–OH has a dielectric constant of 36, which is 3.6 times higher than that of pure PVDF. This enhancement is obviously attributed to the considerably higher dielectric constant of BST in comparison with that of the polymer matrix [18]. Additionally, the dielectric constant of our composites filled with the cube-shaped BST–NPs–OH is much higher than that of the composites filled with spherical fillers [24]. This is because cube-shaped fillers have higher surface area, which is helpful to increase the connectivity of composites. In addition, the samples made with the BST–NPs–OH always have slightly larger dielectric constant than those with the BST–NPs. In addition, the loss of the BST–NPs–OH/PVDF is lower than that of the BST–NPs/PVDF. The result should be attributed to the effect of the surface modification. As discussed above, hydroxyl groups on surface of the BST–NPs–OH facilitated homogenous particle distribution in the polymer matrix. Combining with the results of SEM discussed above, the BST–NPs/PVDF have many defects such as voids in the nanocomposites, which is mean that the air was introduced in nanocomposites. The dielectric constant of air is low. Therefore, the BST–NPs–OH composite films exhibited less agglomeration and defects (such as voids), thus leading to higher dielectric constants [14,25].

**Figure 10:**
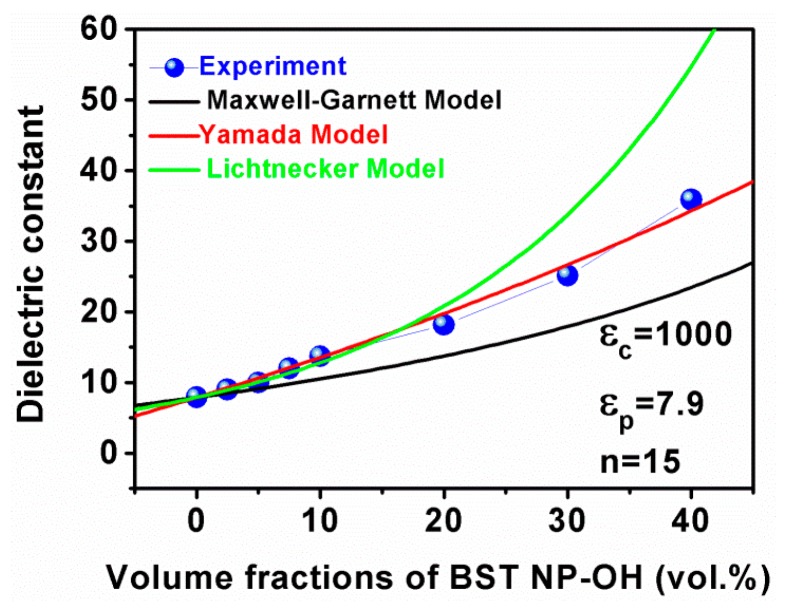
Theoretical and experimental dielectric constants of the composites as a function of content of the BST–NPs–OH powder.

Various theoretical models have been proposed to explain the dielectric behaviors of composite materials. Figure 10 shows experimental and theoretical dielectric constants of the BST–NPs–OH nanocomposites at 1 kHz and room temperature as a function of filler volume fraction, where the theoretical values were predicted by the Maxwell–Garnett model, Lichtenecker model and Yamada model. Maxwell–Garnett model is given by:

(1) $$\varepsilon_{eff}=\varepsilon_{p}\frac{2\varepsilon_{p}+\varepsilon_{c}+2f(\varepsilon_{c}-\varepsilon_{p})}{2\varepsilon_{p}+\varepsilon_{c}-f(\varepsilon_{c}-\varepsilon_{p})}$$

where $f_{c}$ is the volume fraction of fillers, while $\varepsilon_{eff}$, $\varepsilon_{p}$ and $\varepsilon_{c}$ represent the dielectric constants of the nanocomposites, PVDF and BST–NPs, respectively. Lichtenecker model is described as:

(2) $$\ln\varepsilon_{eff}=\ln\varepsilon_{p}+f(1-k)(\ln\varepsilon_{c}-\ln\varepsilon_{p})$$

where k is a shape-dependent parameter. Yamada model is presented as:

(3) $$\varepsilon_{eff}=\varepsilon_{p}[1+\frac{nf_{c}(\varepsilon_{c}-\varepsilon_{p})}{n\varepsilon_{p}+(1-f_{c})(\varepsilon_{c}-\varepsilon_{p})}]$$

where n is a shape-dependent parameter. From Figure 10, it is found that the experimental data are in a harmonious agreement with those given by the Maxwell–Garnett model and Lichtenecker model when the volume fraction of BST–NPs–OH powder was less than 10%. At higher contents of BST–NPs–OH powder, more interfaces were present in the nanocomposites, while these such interfaces are not taken into account by these two models. Therefore, their predictions are deviated from the experimental data of the samples with high filler contents. In contrast, the Yamada model is applicable over the whole concentration range in this study. The shape-dependent parameter of n=15, $\varepsilon_{f}$ = 1000 (reported in the literature [26]) and $\varepsilon_{p}$ = 7.9 are in a good agreement with the measured data. This is because, in the Yamada model, the interactions between the neighboring particles have been taken into account and a shape-dependent parameter has been used. Shape-dependent parameter values related to the geometry of the ceramic particles from 8 to 9.5 have been reported in the literature [27,28]. One reason for this disparity is the difference size of fillers. The ceramic fillers reported in the literature have an average size of about 3 µm [27,28], while the size of BST–NPs fillers in this article is about 110 nm.

**Figure 11:**
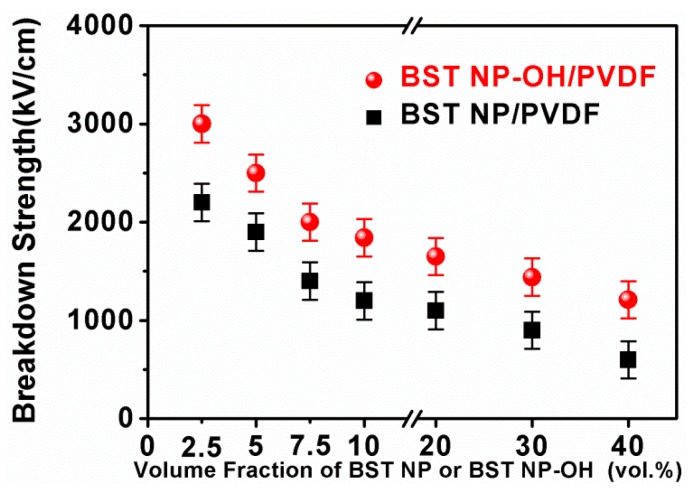
Breakdown strengths the BST–NP/PVDF and BST–NP–OH/PVDF nanocomposites as a function of the filler concentration.

Figure 11 shows breakdown strengths for the nanocomposites as a function of the filler content. The breakdown strength of both nanocomposites monotonically decreases with increasing filler volume fraction. The breakdown strength is strongly influenced by morphology of ceramic fillers, interface areas, agglomerations, increasing air voids, and the large difference of the dielectric constant between the ceramic fillers and the PVDF. This can be generally attributed to the increase in inhomogeneous electrical field, agglomeration and defects in the nanocomposites [29]. When the ceramic fillers are introduced into the polymer matrix, a distortion in the distribution of the electric field is produced, due to the large difference in dielectric properties between the two phases. In this case, the electrical field in the PVDF matrix is much higher than the average electric field. Therefore, overall breakdown strength of the nanocomposites is decreased. On the other hand, the breakdown strength of the polymer–matrix composites could be largely reduced, if agglomerations and defects are formed. As stated earlier, with increasing filler content, particle agglomeration and for the formation of voids cannot be avoided.

Specifically, the nanocomposites with the BST–NPs–OH always have a higher breakdown strength than the BST–NP counterparts. For example, at 40 vol %, the breakdown strength of the nanocomposite with the BST–NPs–OH is 1210 kV/cm, about two times that of the BST–NP sample (605 kV/cm). This observation is attributed the effect of the surface hydroxylation. The surface hydroxylation benefits the homogenous distribution of BST–NPs–OH in the polymer matrix as seen in SEM images and decreases the defects such as voids in the nanocomposites. This factor contributes to the improvement of the breakdown strength of the nanocomposites [30].

**Figure 12:**
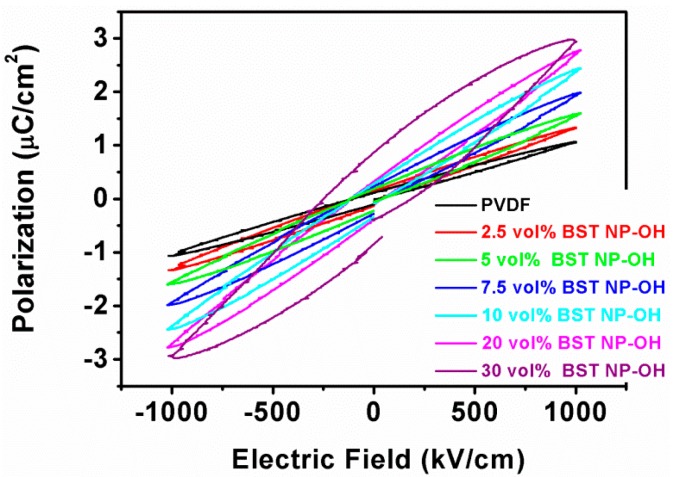
*P-E* loops of the BST–NsP–OH/PVDF nanocomposites with different filler contents.

Energy-storage density is related to not only dielectric constant and breakdown strength, but also the polarization and applied electric field. It is well known that the polarization of ferroelectrics is not linearly dependent on electric field, while both the polarization and dielectric constant of ferroelectric materials are strongly dependent on a variety of external conditions. The energy-storage density of ferroelectric materials can be calculated from the *P-E* loops, with the formula, U=∫EdP (where *E* and *P* are applied electric field and polarization, respectively). Figure 12 shows *P–E* loops at 100 Hz of the BST–NPs–OH/PVDF nanocomposites. At 1000 kV/cm, the polarization of the nancomposites increases constantly with the filler volume fraction and reaches a maximum value of 3.1 μC/cm^2^ at 30 vol %, which should be attributed to the increase in dielectric constant of the nanocomposites. However, the remnant polarization of the composites is also increased with increasing content of BST–NPs–OH. A high remnant polarization means a low energy-storage density, because the integrated area of the *P-E* curve associated with the discharge cycle is decreased.

**Figure 13:**
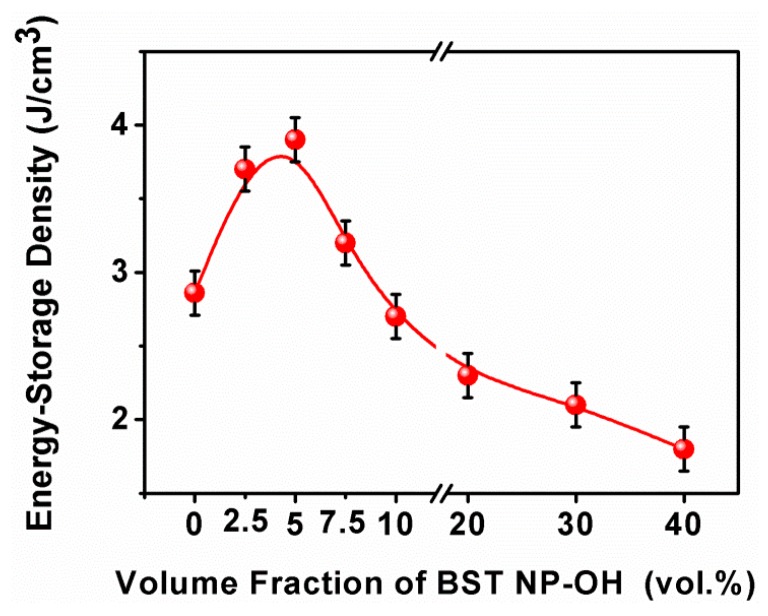
Energy storage density of the BST–NP–OH/PVDF nanocomposites as a function of the filler concentration.

Figure 13 shows room-temperature energy-storage density of the nanocomposites. Obviously, the energy-storage density is strongly dependent on the BST–NPs–OH content, which is maximized at 5 vol %, with a value of 3.9 J/cm^3^ (at 2500 kV/cm). This value is higher than that of the pure PVDF (2.8 J/cm^3^) at 4000 kV/cm.

## 5. Conclusions

Surface hydroxylation of BST–NPs ceramic fillers has a positive effect on dielectric properties, breakdown strength and energy storage densities of the PVDF based nanocomposites, due to the improvement in homogeneity of the nanocomposites. A maximum dielectric constant of 36 (1 kHz) was observed in the BST–NPs–OH sample with a filler concentration of 40 vol %. The sample had a breakdown strength of 1210 kV/cm, two times higher than that the BST–NPs counterpart. A maximal energy density of 3.9 J/cm^3^ was obtained in the composite sample with 5 vol % BST–NPs–OH. It is believed that the finding of this study can be extended to other composites in order to achieve high energy storage density for potential applications in energy storage and power capacitor components.
